# (1,0)-Super Solutions of (*k*,*s*)-CNF Formula

**DOI:** 10.3390/e22020253

**Published:** 2020-02-23

**Authors:** Zufeng Fu, Daoyun Xu, Yongping Wang

**Affiliations:** 1 College of Computer Science and Technology, Guizhou University, Guiyang 550025, China; fuzufeng@outlook.com (Z.F.);; 2 Dep. of Electronics and Information Engineering, Anshun University, Anshun 561000, China; 3 School of Mathematics and Statistics, Guizhou University of Finance and Economics, Guiyang 550025, China

**Keywords:** (*k*,*s*)-CNF, SAT-problem, (1,0)-super solutions

## Abstract

A (1,0)-super solution is a satisfying assignment such that if the value of any one variable is flipped to the opposite value, the new assignment is still a satisfying assignment. Namely, every clause must contain at least two satisfied literals. Because of its robustness, super solutions are concerned in combinatorial optimization problems and decision problems. In this paper, we investigate the existence conditions of the (1,0)-super solution of (k,s)-CNF formula, and give a reduction method that transform from *k*-SAT to (1,0)-(k+1,s)-SAT if there is a (k+1,s)-CNF formula without a (1,0)-super solution. Here, (k,s)-CNF is a subclass of CNF in which each clause has exactly *k* distinct literals, and each variable occurs at most *s* times. (1,0)-(k,s)-SAT is a problem to decide whether a (k,s)-CNF formula has a (1,0)-super solution. We prove that for k>3, if there exists a (k,s)-CNF formula without a (1,0)-super solution, (1,0)-(k,s)-SAT is NP-complete. We show that for k>3, there is a critical function φ(k) such that every (k,s)-CNF formula has a (1,0)-super solution for s≤φ(k) and (1,0)-(k,s)-SAT is NP-complete for s>φ(k). We further show some properties of the critical function φ(k).

## 1. Introduction

In combinatorial optimization problems and decision problems, the robustness of solutions is a valuable property. A robust solution is not sensitive to small changes in dynamic and uncertain environments, and guarantees the existence of a small set of repairs when the future changes in a small way. R. Weigel and C. Bliek in [1] introduced the concept of fault tolerant solutions to constraint programming (CP) and presented some methods to find fault tolerant solutions. A. Parkes etc. in [2] introduced Supermodels to measure solution robustness in propositional satisfiability (SAT). Fowler and Brown in [3] focused on finding robust partial solutions to a dynamically changing problem. In [4], the concept of (a,b)-super solution was introduced in order to depicts formally the robustness of a solution. If the values assigned to any *a* variables by a solution are no longer available, the solution can be repaired by changing values of *a* variables and at most *b* other variables. Then the solution is an (*a*,*b*)-super solution. For example, the solution <0,1,1> is a (1,1)-super solution of the formula $F=\left(x_{1}\vee x_{2}\vee x_{3}\right)\wedge \left(\neg x_{1}\vee \neg x_{2}\vee \neg x_{3}\right)$. But the solution is not a (1,0)-super solution, because if $x_{1}$ loses the value 0, we must change at least one of $x_{2}$ and $x_{3}$ to repair the solution.

Encoding into a CNF formula is a common way to solve a practical problem. These CNF formulas often have some regular structures. A (*a*,*b*)-super solution of a CNF formula with special structure is undoubtedly of realistic significance. Therefore, we focus on some special (1,0)-SAT problems that are some decision problems whether a CNF formula with special structure has a (1,0)-super solution. In this paper, we investigate the existence conditions of the (1,0)-super solution of a CNF formula with special structure and the NP-completeness of (1,0)-(k,s)-SAT. We also show that for k>3, (1,0)-(*k*,*s*)-SAT has a SAT-UNSAT transition phenomenon and investigate the characteristics of the transition phenomenon.

## 2. Related Works

An (*a*,*b*)-super solution is a generalization of Supermodels in SAT problems. Some algorithms of finding super solutions were presented in [5,6,7]. A weighted super solution framework in [8] was presented for the lack of expressive of the classical super solutions framework, and has been successfully used to find robust solutions to combinatorial auctions.

Specially, a (1,0)-super solution is a satisfying assignment such that if any one variable is flipped to the opposite value, the new assignment is still a satisfying assignment. In other words, a (1,0)-super solution of a CNF formula must satisfy at least two liters of every clause. The decision problem whether a CNF formulas has a (1,0)-super solution is denoted as (1,0)-SAT. (1,0)-*k*-SAT is the special version of (1,0)-SAT where each clause has exactly *k* distinct literals. Zhang P in [9] proved that (1,0)-*k*-SAT is in P for k≤3 and in NP-complete for k≥4. Besides, it was proved that a random (1,0)-3-SAT are satisfiable with high probability for Constrained Density (the clause-to-variable ratio) α<1/3, and not satisfiable with high probability for α>1/3. For k≥4, the upper bound of the phase transition point is 2kln2/(k+1). By using an enhanced weighting scheme, Zhou G in [10] obtained a better lower bound of the phase transition point.

(1,0)-3-SAT problem is in P. This shows that there is a polynomial time algorithm to decide whether a 3-CNF formula has a (1,0)-super solution. For k≥4, (1,0)-*k*-SAT is NP-complete. It implies that for k≥4, (1,0)-*k*-SAT can not be solved in polynomial time. What happens if the number of occurrences of each variable is limited? So we expand (1,0)-*k*-SAT to (1,0)-(k,s)-SAT, (1,0)-regular (k,s)-SAT and (1,0)-*d*-regular (k,s)-SAT. They correspond to (k,s)-CNF formula, regular (k,s)-CNF formula and *d*-regular (k,s)-CNF formula, respectively. Here a (k,s)-CNF formula is a CNF formula with exactly *k* distinct literals per clause and at most *s* occurrences of each variable. A regular (*k*,*s*)-CNF formula is a *k*-CNF formula, in which each variable occurs exactly in *s* clauses. A *d*-regular (k,s)-CNF formula also requires that the absolute value of the difference between positive and negative occurrences of each variable is no more than nonnegative *d*. The NP-completeness of the special SAT problems worth further study.

Kratochvíl in [11] pointed out that for k≥3, there exists a critical function f(k) such that

(i) for s≤f(k), every formula in (k,s)-CNF is satisfiable;

(ii) for s≥f(k)+1, (k,s)-SAT is already NP-complete.

f(k) represents also the maximum *s* such that all (k,s)-CNF formulas are satisfiable. The critical function f(k) has been studied extensively. The exact values of f(k) are only known for k=3 and k=4, because f(3)=3 and f(4)=4 were shown in [11]. In [12,13,14,15], it was showed that the best known upper and lower bounds of f(k) are described as follows

5≤f(5)≤7,7≤f(6)≤11,13≤f(7)≤17,24≤f(8)≤29,41≤f(9)≤51.

In this study, we give a polynomial time reduction that transforms *k*-SAT to (1,0)-(k+1,s)-SAT if an unsatisfiable instance of (1,0)-(k+1,s)-SAT exists. We prove that for k>3, if there exists a (k,s)-CNF formula without a (1,0)-super solution, then (1,0)-(k,s)-SAT is NP-complete. This study shows that for k>3, there is a critical function φ(k) such that

(i) every (k,s)-CNF formula has a (1,0)-super solution for s≤φ(k) and

(ii) (1,0)-(k,s)-SAT is NP-complete for s>φ(k).

That is to say, for k>3, (1,0)-(k,s)-SAT has the SAT-UNSAT transition phenomenon. We find that the critical function φ(k) is an increasing function and for k>3, φ(k)≥2 and φ(k+1)≤2φ(k)+1.

## 3. Notations

A literal is a propositional variable *x* or a negated propositional variable ¬x. *x* is called a positive literal, and ¬x is called a negative literal. A pure literal is a literal whose complement does not occur in a formula. A clause *C* is a disjunction of literals, $C=L_{1}\vee L_{2}\vee \ldots \vee L_{k}$ or $C=L_{1},L_{2},\ldots ,L_{k}$. A CNF formula *F* is a conjunction of clauses, $F=C_{1}\wedge C_{2}\wedge \ldots \wedge C_{m}$ or $F=[C_{1},C_{2},\ldots ,C_{m}]$. var(F) is denoted as the set of variables occurring in a formula *F*, and #var(F) refers to the number of variables. #cl(F) is denoted as the number of clauses of *F*. Occ(F,x) refers to the number of occurrences of a variable *x* in *F*.

If the formulas Φ and Ψ are either satisfiable at the same time or not, they are called SAT-equivalents. A formula $F^{\prime }$ is called the disjoint copy of a CNF formula *F*, if $F^{\prime }$ is a copy of *F* but their variable sets are disjoint. We divide variables into forced variables or unforced variables. A forced variable is a variable forced a same value by all satisfying assignments of a formula.

A satisfying assignment is also called a solution. The set of solutions of a CNF formula *F* is denoted by SAT(F). The Hamming distance $H(\tau_{1},\tau_{2})$ between two assignments $\tau_{1}$ and $\tau_{2}$ is the Hamming distance between the two corresponding vectors in ${\left\{0,1\right\}}^{n}$, i.e.,

$$H\left(\tau_{1},\tau_{2}\right)=\left|\left\{1\leq i\leq n:\tau_{1}\left(x_{i}\right)\neq \tau_{2}\left(x_{i}\right)\right\}\right|.$$

The *d*-Hamming neighbourhood $N_{d}\left(\tau_{1}\right)$ of an assignment $\tau_{1}$ is defined as

$$N_{d}\left(\tau_{1}\right)=\left\{\tau \in {\left\{0,1\right\}}^{n}:H\left(\tau_{1},\tau \right)\leq d\right\}.$$

An (a,b)-super solution τ of a SAT instance *F* is a satisfying assignment such that for any σ with H(τ,σ)=a, we have $N_{b}\left(\sigma \right)⋂SAT\left(F\right)\neq \emptyset$.

**Definition** **1.** *A CNF formula is called a regular (k,s)-CNF, if each clause of the formula has exactly k distinct literals and each variable occurs exactly in s clauses. A d-regular (k,s)-CNF formula is a regular (k,s)-CNF formula, in which the absolute value of the difference between positive and negative occurrences of every variable is at most a nonnegative integer d.*

**Definition** **2.** *For each k≥3, f(k) is defined as the maximum s such that all (k,s)-CNF formulas are satisfiable, and φ(k) is defined as the maximum s such that all (k,s)-CNF formulas must have a (1,0)-super solution.*

**Definition** **3.** *A (k,s)-CNF formula *Ψ* is a forced-(k,s)-CNF formula if*

*(i) there exist two variables x,y that Occ(Ψ,x)=1 and Occ(Ψ,y)<s;*

*(ii) *Ψ* has a (1,0)-super solution and for any (1,0)-super solution τ of *Ψ*, it holds that τ(x)=τ(y)=true.*

**Definition** **4.** *The projection π(C) of a k-clause $C=l_{1}\vee l_{2}\vee \ldots \vee l_{k}$ is defined as $\pi \left(C\right)=\wedge_{i=1}^{k}\left(\vee_{j\neq i}l_{j}\right)$. The projection of a CNF formula $F=C_{1}\wedge \cdots \wedge C_{m}$ is defined as $\pi \left(F\right)=\wedge_{i=1}^{m}\left(\pi \left(C_{i}\right)\right)$.*

**Lemma** **1** ([9])**.** *An assignment is a (1,0)-super solution of F if and only if it satisfies π(F).*

**Lemma** **2** ([11])**.** *If k≥3 and s are such that an unsatisfiable (k,s)-CNF formula exists, then (k,s)-SAT is NP-complete.*

**Lemma** **3** ([11])**.** *The critical function f(k) is strictly increasing.*

**Lemma** **4** ([16])**.** *An instance of SAT is satisfiable if:*

*(i) all of its clauses contain more than one variable, and*

*(ii) each variable appears exactly once complemented and once uncomplemented.*

**Lemma** **5.** *If the representation matrix of a formula F is*

$$\begin{array}{c} x_{1}\\ x_{2}\\ \vdots \\ \vdots \\ x_{n-1}\\ x_{n} \end{array}\left(\begin{array}{ccccc} + & \; & \; & \; & -\\ - & + & \; & \; & \;\\ \; & - & \; & \; & \;\\ \; & \; & \ddots & \; & \;\\ \; & \; & \; & + & \;\\ \; & \; & \; & - & + \end{array}\right),$$

*then the formula is satisfiable and every satisfying assignment forces all variables to a same value.*

Here, if an element $a_{ij}$ of the matrix is +, the *i*th variable occurs positively in the *j*th clause; if $a_{ij}=-$, the *i*th variable occurs negatively in the *j*th clause; if $a_{ij}=0$, otherwise. The formula *F* expresses a cyclic of implication

$$x_{1}\to x_{2}\to \ldots \to x_{n-1}\to x_{n}\to x_{1}.$$

Therefore, it is easy to obtain Lemma 5.

## 4. The Existence of (1,0)-Super Solution of (k,s)-CNF Formula

A (1,0)-super solution has certain robustness, but some CNF formula with special structures may not have a (1,0)-super solution.

**Theorem** **1.** *Every regular (3,2)-CNF Formula without pure literals does not have a (1,0)-super solution.*

**Proof.**  If a CNF formula *F* is a regular (3,2)-CNF without pure literals, then each clause has exactly 3 distinct literals and each variable occurs exactly 2 times (one negative occurrence and another positive occurrence). Suppose, *F* has 3n variables, so it has 2n clauses and 6n literals. Because every assignment only satisfies half of occurrence of each variable, it only satisfies 3n literals of *F*. However, a (1,0)-super solution must satisfy at least two literals of every clause. That is to say, a (1,0)-super solution must satisfy at least 4n literals of the formula *F*. Clearly, there is not a (1,0)-super solution. Therefore, no regular (3,2)-CNF Formula without pure literals has a (1,0)-super solution. □

By bounding the difference between positive and negative occurrences of every variable, we find other CNF formulas without a (1,0)-super solution.

**Corollary** **1.** *For s≥4, every 1-regular (3,s)-CNF formula does not have a (1,0)-super solution.*

**Proof.**  Let *F* is a 1-regular (3,s)-CNF formula. By definition, each clause has exactly 3 distinct literals, and each variable occurs exactly *s* times, and the absolute value of the difference between positive and negative occurrences of each variable is at most 1. Suppose *F* has 3n variables, so it must have sn clauses and 3sn literals. Every assignment only satisfies 3n⌈s/2⌉ literals of *F*, but every (1,0)-super solution must satisfy at least 2sn literals of *F*. For s≥4, we obtain 2sn>3n⌈s/2⌉. Therefore, for s≥4, no 1-regular (3,s)-CNF Formula has a (1,0)-super solution. □

Now we consider an arbitrary 1-regular (3,3)-CNF formula *F*. Suppose *F* has 3n variables, so it must have 3n clauses and 3n literals. Every variable occur exactly in three clauses of *F* (one negative occurrence and two positive occurrence, or two negative occurrence and one positive occurrence). Because every (1,0)-super solution must satisfy at least 6n literals of *F*, only an assignment τ that satisfy every literal with maximum number of occurrences can satisfy at least 6n literals of *F*. In other words, if τ is not a (1,0)-super solution, the formula *F* does not have a (1,0)-super solution. That is, if the formula has a (1,0)-super solution, the (1,0)-super solution must be τ. For example, F={x∨¬y∨¬z,¬x∨¬y∨z,x∨y∨z}. Only one assignment <1,0,1> is a (1,0)-super solution.

Moreover, we also find some (k,s)-CNF formulas with other special structures that must have a (1,0)-super solution.

**Theorem** **2.** *Every regular (4,2)-CNF formula must have a (1,0)-super solution.*

**Proof.**  Suppose Ψ is a regular (4,2)-CNF formula with *m* clauses. By definition, each clause has exactly 4 distinct literals, and each variable occurs exactly in 2 clauses (one negative occurrence and another positive occurrence). We divide each clauses $c_{i},1\leq i\leq m$ of Ψ into two equal segments $c_{i1}$ and $c_{i2}$, and construct a CNF formula Φ by using all divided segments. The dividing processes does not change the number of occurrences of every variable. Clearly, Φ is a regular (2,2)-CNF formula. As a nice application of Hall’s Marriage Theorem, it is proved that any one of regular (2,2)-CNF formulas is satisfiable in [16]. Suppose, an assignment τ satisfies the formula Φ. It means that τ must satisfy each pair of $c_{i1}$ and $c_{i2}$, for 1≤i≤m. In other words, both $c_{i1}$ and $c_{i2}$ have at least one literal satisfied. Therefore, there are at least two literals satisfied by the assignment τ in each clause $c_{i}$ of Ψ. That is to say, the assignment τ is a (1,0)-super solution of Ψ. □

Theorem 2 can be considered as an extension of Hall’s Marriage Theorem. Suppose we have a finite set of single men and women. If each man is attracted by two women and each woman is attracted by four man, then there must be a way that each woman could be married with two men at the same time.

Using Lemma 4, it is easy to obtain the following corollaries by using the proof method of Theorem 2.

**Corollary** **2.** *For k≥4, each (k,2)-CNF formula must have a (1,0)-super solution.*

**Corollary** **3.** *A CNF formula must have a (1,0)-super solution if:*

*(i) each clause has at least 4 distinct variables and*

*(ii) each variable occurs exactly in two clauses(one negative occurrence and another positive occurrence).*

For the formula in Corollary 3, all clauses may not have the same number of variables.

**Theorem** **3.** *All (9,3)-CNF formulas and (10,4)-CNF formulas must have a (1,0)-super solution.*

**Proof.**  Suppose Ψ is a (9,3)-CNF formula. Define $\Phi =\pi \left(\Psi \right)=\wedge_{C\in \Psi }\left(\pi \left(C\right)\right)$. Because $\pi \left(C\right)=\wedge_{i=1}^{9}\left(\vee_{j\neq i}l_{j}\right)$, the formula Φ is a 8-CNF formula and the number of occurrences of every variable was enlarged by 8 times in π(C). That is to say, every variable of Φ occurs in at most 24 clauses. Because 24≤f(8)≤29 in [15], all (8,24)-CNF formulas are satisfiable. Therefore, the formula Φ is satisfiable. Lemma 1 entails that the formula Ψ must have a (1,0)-super solution. It means that all (9,3)-CNF formulas must have a (1,0)-super solution.

Similarly, we get that all (10,4)-CNF formulas must have a (1,0)-super solution by 41≤f(9)≤51 in [15]. □

In [11], it has been shown that for s≤f(x), all (k,s)-CNF formulas must be satisfiable. By Theorem 3, we get for s≤3, all (9,s)-CNF formulas must have a (1,0)-super solution. Similarly, for s≤4, all (10,s)-CNF formulas must have a (1,0)-super solution.

## 5. The NP-Completeness of (1,0)-(k,s)-SAT

In this section we study in what conditions (1,0)-(k,s)-SAT is NP-complete. Here, (1,0)-(k,s)-SAT refers to a decision problem whether a (k,s)-CNF formula has a (1,0)-super solution.

**Theorem** **4.** *For k≥3, if (k,s)-SAT is NP-complete, then (1,0)-(k+1,s)-SAT is NP-complete.*

**Proof.**  We will present a polynomial time reduction method from (k,s)-SAT to (1,0)-(k+1,s)-SAT. Let Ψ is a (k,s)-CNF formula. Without loss of generality, we suppose that Ψ has m>0 clauses. The reduction method has three steps, which are described as follows.

**Step 1** Let $f=C_{1}\wedge C_{2}$. Here

$$C_{1}=x_{1}\vee x_{2}\vee \neg x_{3}\vee \neg x_{4}\vee x_{5}\vee \ldots \vee x_{k+1},C_{2}=\neg x_{1}\vee \neg x_{2}\vee x_{3}\vee x_{4}\vee \neg x_{5}\vee \ldots \vee \neg x_{k+1},$$

$x_{1},x_{2},\ldots ,x_{k+1}$ are some new variables which do not occur in Ψ. Clearly, an assignment forcing $x_{1},x_{2},x_{3},x_{4}$ to *true* is a (1,0)-super solution of the formula *f*.

**Step 2** Let $f_{i},1\leq i\leq m$ be disjoint copies of the formula *f* with the variables $x_{j},1\leq j\leq k+1$ of *f* being renamed as $x_{i,j}$ in $f_{i}$. Let $X=\{x_{i,j},1\leq i\leq m,1\leq j\leq k+1\}$ and $\Psi_{1}=\wedge_{C_{i}\in \Psi }\left(C_{i}\vee x_{i,1}\right)$.

**Step 3** We construct the formula $\Phi =\Psi_{1}\wedge f_{1}\wedge f_{2}\wedge \ldots \wedge f_{m}$.

Obviously, every clause of Φ has exactly k+1 distinct literals and every variable of Φ occurs in at most *s* clauses. So Φ is a (k+1,s)-CNF formula. Next, we will prove that Ψ is satisfiable if and only if Φ has a (1,0)-super solution.

It is supposed that Ψ is satisfiable. Let τ is a satisfying assignment of Ψ. A truth assignment $\tau^{\prime }$ is defined by

$$\tau^{\prime }\left(v\right)=\left\{\begin{array}{cc} \tau (v), & if\;v\;\in \;var(\Psi )\\ true, & if\;v\;\in var(f_{i}),1\leq i\leq m \end{array}\right.$$

Obviously, the truth assignment $\tau^{\prime }$ satisfies at least two literals of every clause of Φ. That is to say, $\tau^{\prime }$ is a (1,0)-super solution of Φ.

It is supposed that τ is a (1,0)-super solution of Φ. That is, τ satisfies at least two literals of every clause of $\Psi_{1}$. Because $\Psi_{1}=\wedge_{C_{i}\in \Psi }\left(C_{i}\vee x_{i,1}\right)$, every clause $C_{i}\vee x_{i,1}$ must have two satisfied literals. It is clear that τ satisfies at least one literal of $C_{i}\in \Psi$. In other words, τ is a satisfying assignment of Ψ, and Ψ is satisfiable.

For k≥3, if (k,s)-SAT problem is NP-complete, it can be obtained that (1,0)-(k+1,s)-SAT is NP-complete. □

The critical function f(k) of (k,s)-SAT indicate that if (k,s)-SAT is an NP-complete problem, then for any m≥0, (k,s+m)-SAT is NP-complete. By Theorem 4, if (k,s)-SAT is an NP-complete problem, then for any m≥0, (1,0)-(k+1,s+m)-SAT is NP-complete.

**Corollary** **4.** *If k≥3 and s are such that an unsatisfiable (k,s)-CNF formula exists, (1,0)-(k+1,s)-SAT is NP-complete.*

**Proof.**  The statement follows directly from Lemma 2 and Theorem 4. □

By Hall’s Marriage Theorem, (4,4)-CNF and (5,5)-CNF are satisfiable. But (1,0)-(4,4)-SAT and (1,0)-(5,5)-SAT are NP-complete because (3,4)-SAT and (4,5)-SAT are NP-complete.

Next, we make some modifications of the reduction method in the proof of Theorem 4. We introduce a sufficient number of new variables, add some new clauses to make up the gap of the occurrence number of every variable, and guarantee that every new clause have at least a positive occurrence of two new variables (so, the new clauses must have a (1,0)-super solution). Because s>k≥3 (for an unsatisfiable (k,s)-CNF formula exists), the method is feasible. So the reduction method is modified to transform (k,s)-SAT to (1,0)-regular (k+1,s)-SAT. Therefore we get Corollary 5.

**Corollary** **5.** *If k≥3 and s are such that an unsatisfiable (k,s)-CNF formula exists, (1,0)-regular (k+1,s)-SAT is NP-complete.*

Here, (1,0)-regular (k,s)-SAT refers to a decision problem whether a regular (k,s)-CNF formula has a (1,0)-super solution. Because an unsatisfiable (3,4)-CNF formula exists, (1,0)-regular (4,4)-SAT is NP-complete.

**Lemma** **6.** *If k≥3 and s are such that a satisfiable (k,s)-CNF formula does not have a (1,0)-super solution, then a forced-(k,s)-CNF formula exists.*

**Proof.**  We will show a way to construct a forced-(k,s)-CNF formula.

It is supposed that a (k,s)-CNF formula Ψ is satisfiable but does not have a (1,0)-super solution. Let τ is a satisfying assignment of Ψ. Clearly, τ is not a (1,0)-super solution of Ψ. That is, there must have some clauses in which only one literal is satisfied by τ. Next, we will seek for a *key-literal*. A *key-literal* refers to a literal which is flipped to make the formula without a (1,0)-super solution have a (1,0)-super solution. First we flip a unsatisfied literal of any one clause with only one satisfied literal. If the formula still does not have a (1,0)-super solution, then we flip a unsatisfied literal of other clause with only one satisfied literal, until the formula has a (1,0)-super solution. When the formula has a (1,0)-super solution, the flipped literal is a *key-literal*.

Without loss of generality, it is supposed that ¬y in a clause *c* is a *key-literal*. Because ¬y in the clause *c* of Ψ is a key-literal, it means that any one of assignments satisfies only one literal of the clause *c* of Ψ, and flipping the literal ¬y (only in the clause *c*) can make Ψ have a (1,0)-super solution. Clearly, every (1,0)-super solution satisfies only one literal of the original clause *c* and only two literals of the flipped clause. This also indicates that all (1,0)-super solutions satisfy the literal *y* (the complement of the literal ¬y).

The clause set of Ψ is denoted *C*. Let $C_{1}=\left(C-c\right)\cup c_{1}$, $c_{1}=\left(c-\neg y\right)\cup x$. Here, *x* is a new variable which does not occur in Ψ. Define $\Phi =\wedge_{c\in C_{1}}\left(c\right)$. Obviously, Φ must have a (1,0)-super solution and every (1,0)-super solution of Φ must force x,y to true (if an assignment forces *x*false, then the assignment can not satisfy at least two literals of $c_{1}$). Therefore, Φ is a forced-(k,s)-CNF formula. □

**Theorem** **5.** *If k≥3 and s are such that an unsatisfiable instance of (1,0)-(k+1,s)-SAT exists, then (1,0)-(k+1,s)-SAT is NP-complete.*

**Proof.**  It is supposed that a (k+1,s)-CNF formula *F* does not have a (1,0)-super solution. There are two cases to consider.

**Case 1:** the formula *F* is unsatisfiable. This illustrates that f(k+1)<s. By Lemma 3, f(k)<s. That is to say, there exists an unsatisfiable instance of (k,s)-SAT. Corollary 5 entails that (1,0)-(k+1,s)-SAT is NP-complete.

**Case 2:** the formula *F* is satisfiable but does not have a (1,0)-super solution. Lemma 6 entails that we can construct a forced-(k+1,s)-CNF formula Φ. Next we present a polynomial time reduction method from an instance of *k*-SAT to an instance of (1,0)-(k+1,s)-SAT.

Let Ψ is a *k*-CNF formula with *m* clauses. Obviously, Ψ has mk literals. The reduction method has four steps, which are described as follows.

**Step 1** Let $\Phi_{ij},1\leq i\leq mk,1\leq j\leq k-2$ be disjoint copies of the formula Φ with the variables x,y of Φ being renamed as $x_{i,j},y_{i,j}$ in $\Phi_{ij}$. These formulas and the formula Ψ have pairwise disjoint sets of variables. Let $\Psi_{1}=\wedge_{1\leq i\leq mk}\wedge_{1\leq j\leq k-2}\Phi_{ij}$ and $X=\left\{x_{i,j}\right\}\left(1\leq i\leq mk,1\leq j\leq k-2\right)$.

**Step 2** We introduce a new variable set $Z=\left\{z_{i,j}\right\}\left(1\leq i\leq m,1\leq j\leq k\right)$ to replace mk literals in Ψ in order to construct a new formula $\Psi_{2}$.

$$\Psi_{2}=\wedge_{1\leq i\leq m}\left(y_{i,1}\vee \left(\vee_{1\leq j\leq k}L_{i,j}^{\prime }\right)\right),\;L_{i,j}^{\prime }=\left\{\begin{array}{cc} z_{i,j}, & if\;L_{i,j}=z\\ \neg z_{i,j}, & if\;L_{i,j}=\neg z \end{array}\right.,\;z\in var\left(\Psi \right).$$

Here, $L_{i,j}$ is the *j*th literal of the *i*th clause of Ψ.

**Step 3** Let $\Psi_{3}={⋀}_{1\leq i\leq mk}d_{i}$, and $d_{i}=z_{i}\vee \neg z_{j}\vee \neg x_{i,1}\vee \neg x_{i,2}\vee \ldots \vee \neg x_{i,k-2}\vee x_{j,1}$. Here $z_{i},z_{j}\in Z$ and the variables of *Z* are sorted by their subscripts. In addition, $z_{i}$ replaces a variable *v* in Ψ and $z_{j}$ be the next variable of *Z* which replaces *v* (if $z_{i}$ is the last variable in the variable set *Z* which replaces the variable *v*, $z_{j}$ is set to be the first).

**Step 4** We construct the formula $\Psi^{\prime }=\left\{\Psi_{1},\Psi_{2},\Psi_{3}\right\}$.

Using Corollary 2, if k≥3 and *s* are such that an unsatisfiable instance of (1,0)-(k+1,*s*)-SAT exists, then s≥3. In $\Psi^{\prime }$, every variable of *Z* occurs in three clauses and every variable of *X* occurs in two clauses. Obviously, the formula $\Psi^{\prime }$ is a (k+1,s)-CNF formula and can be constructed in polynomial time. Next, we will prove that $\Psi^{\prime }$ has a (1,0)-super solution if and only if Ψ is satisfiable.

It is assumed that an assignment τ is a (1,0)-super solution of $\Psi^{\prime }$. This suggests that τ satisfies at least two literals of every clause of $\Psi^{\prime }$. Because every $\Phi_{ij}$ is a forced-(k+1,s)-CNF formula, we get $\tau \left(x_{i,j}\right)=true,\tau \left(y_{i,j}\right)=true,\neg \tau \left(x_{i,j}\right)=false,1\leq i\leq mk,1\leq j\leq k-2$. We substitute $\tau (x_{i,j})$ into $\Psi_{3}$, and simplify $\Psi_{3}$. Using Lemma 5, it can be obtained that any one (1,0)-super solution enable the simplified $\Psi_{3}$ to express *n* cyclic of implication. That is, if $z_{i}$ and $z_{j}$ replace the same variable of Ψ, $\tau \left(z_{i}\right)=\tau \left(z_{j}\right)$. Therefore, we define a truth assignment $\tau^{\prime }$ by

$$\tau^{\prime }\left(v\right)=\tau \left(z\right),\;if\;a\;variable\;v\;of\;\Psi \;is\;replaced\;with\;a\;variable\;z\;in\;Z.$$

Because the (1,0)-super solution τ satisfies at least two literals of every clause of $\Psi_{2}$, the assignment $\tau^{\prime }$ satisfies at least one literal of every clause of Ψ. Thus, Ψ is satisfiable.

It is assumed that Ψ is satisfied by a truth assignment τ over var(Ψ) and a truth assignment $\tau_{i,j}$ is a (1,0)-super solution of $\Phi_{i,j},1\leq i\leq mk,1\leq j\leq k-2$. A truth assignment $\tau^{\prime }$ is defined by

$$\tau^{\prime }\left(v\right)=\left\{\begin{array}{cc} \tau (x), & if\;v\in Z\;and\;a\;variable\;x\;of\;var(\Psi )\;is\;replaced\;with\;v\\ \tau_{i,j}\left(v\right), & if\;v\in var(\Phi_{ij}) \end{array}\right.$$

Because $\tau_{i,j}$ is a (1,0)-super solution, $\tau_{i,j}$ satisfies at lest two literals of every clause of $\Phi_{i,j}$. Therefore, $\tau^{\prime }$ satisfies at least two literals of every clause of $\Psi_{1}$. Because each $\Phi_{i,j}$ is a forced-(k+1,s)-CNF formula, we get $\tau^{\prime }\left(x_{i,j}\right)=true,\tau^{\prime }\left(y_{i,j}\right)=true,\neg \tau^{\prime }\left(x_{i,j}\right)=false,1\leq i\leq mk,1\leq j\leq k-2$. Because τ satisfies at least one literal of every clause of Ψ, $\tau^{\prime }$ satisfies at least two literals of every clause of $\Psi_{2}$ and $\Psi_{3}$. Thus, $\tau^{\prime }$ is a (1,0)-super solution of $\Psi^{\prime }$.

For k≥3, Because *k*-SAT is NP-complete, (1,0)-(k+1,s)-SAT is NP-complete. □

## 6. The Transition Phenomenon of (1,0)-(k,s)-SAT

In [11], it is proved that for k≥3, there is a transition phenomenon of (k,s)-SAT. We will show a transition phenomenon of (1,0)-(k,s)-SAT.

**Theorem** **6.** *For k>3, there is a critical function φ(k) such that*

*(i) every (k,s)-CNF formula has a (1,0)-super solution for s≤φ(k) and*

*(ii) (1,0)-(k,s)-SAT is NP-complete for s>φ(k).*

**Proof.**  The statement follows directly from Corollary 3 and Theorem 5. □

The critical function φ(k) represents also the maximum *s* such that all (k,s)-CNF formulas have a (1,0)-super solution. Although we don’t know whether φ(k) is computable, it is easy to obtain that the following two corollary by Corollary 2 and Theorem 3.

**Corollary** **6.** *For k>3, φ(k)≥2.*

**Corollary** **7.** *φ(9)≥3, φ(10)≥4.*

In [11], it is showed that f(k) is a strictly increasing function. Next, we will prove that φ(k) is a an increasing function.

**Theorem** **7.** *φ(k+1)≥φ(k).*

**Proof.**  Let Φ is a (k+1,φ(k+1)+1)-CNF formula without a (1,0)-super solution. Obviously, every formula obtained from Φ by deleting some variables from some clauses (while retaining the occurrences in other clauses) still does not have a (1,0)-super solution. Now we let Ψ be a formula obtained from Φ by deleting a arbitrary variable from every clause. Consequently, Ψ is a (k,φ(k+1)+1)-CNF formula without a (1,0)-super solution and φ(k+1)≥φ(k) follows. □

**Theorem** **8.** *φ(k+1)≤2φ(k)+1.*

**Proof.**  It is assumed that Φ is a (k,φ(k)+1)-CNF formula without a (1,0)-super solution and has *m* clauses. We introduce a new variable set $Z=\left\{z_{i}\right\}\left(1\leq i\leq m\right)$. Let

$$\Psi =\wedge_{1\leq i\leq m}\left(\left(z_{i}\vee C_{i}\right)\wedge \left(\neg z_{i}\vee C_{i}\right)\right),C_{i}\in \Phi .$$

Obviously, Ψ is a (k+1,2(φ(k)+1))-CNF formula without a (1,0)-super solution. Consequently, φ(k+1)≤2φ(k)+1. □

## 7. Conclusions

We study the decision problems of (1,0)-super satisfiability of (k,s)-CNF, and prove that the problem has a sudden jump from triviality to NP-completeness for every k>3. In other words, there is a critical function φ(k) such that every (k,s)-CNF formula has a (1,0)-super solution for s≤φ(k) and (1,0)-(k,s)-SAT is NP-complete for s>φ(k). The research on the nature of (1,0)-SAT with regular structure has direct meaning to design the best search algorithm for finding a super solution.

Although we obtain some results about φ(k), we are still left with some questions. For example, it is not known whether φ(k) is computable. Since typical formulas arising in practice have clauses of small width, it is interesting to know the exact values of φ(k) for small *k*. Besides, for larger values of *k*, the upper and lower bounds of φ(k) should be concerned.
